# Mast cells: key players in digestive system tumors and their interactions with immune cells

**DOI:** 10.1038/s41420-024-02258-y

**Published:** 2025-01-15

**Authors:** Feihong Shu, Jie Yu, Youjia Liu, Fang Wang, Guoyou Gou, Min Wen, Chen Luo, Xianmin Lu, Yanxia Hu, Qian Du, Jingyu Xu, Rui Xie

**Affiliations:** 1 Department of Endoscopy and Digestive System, Guizhou Provincial People’s Hospital, Guiyang, China; 2 Zunyi Medical University, Zunyi, Guizhou China; 3 Guizhou Medical University, Guiyang, Guizhou China

**Keywords:** Cancer microenvironment, Outcomes research

## Abstract

Mast cells (MCs) are critical components of both innate and adaptive immune processes. They play a significant role in protecting human health and in the pathophysiology of various illnesses, including allergies, cardiovascular diseases and autoimmune diseases. Recent studies in tumor-related research have demonstrated that mast cells exert a substantial influence on tumor cell behavior and the tumor microenvironment, exhibiting both pro- and anti-tumor effects. Specifically, mast cells not only secrete mediators related to pro-tumor function such as trypsin-like enzymes, chymotrypsin, vascular endothelial cell growth factor and histamine, but also mediators related to anti-tumor progression such as cystatin C and IL-17F. This dual role of mast cells renders them an under-recognized but very promising target for tumor immunotherapy. Digestive system tumors, characterized by high morbidity and associated mortality rates globally, are increasingly recognized as a significant healthcare burden. This paper examines the influence of mast cell-derived mediators on the development of tumors in the digestive system. It also explores the prognostic significance of mast cells in patients with various gastrointestinal cancers at different stages of the disease. Additionally, the article investigates the interactions between mast cells and immune cells, as well as the potential relationships among intratumoral bacteria, immune cells, and mast cell within digestive system microenvironment. The aim is to propose new strategies for the immunotherapy of digestive system tumors by targeting mast cells.

## Facts

- Mast cells, as controversial cells in the tumor microenvironment, have dual characteristics of promoting tumor progression and inhibiting tumor progression.
- The soluble mediators secreted by mast cells and their crosstalk with immune cells can affect the evolution of digestive system tumors.
- There is a connection between intratumoral bacteria and immune cells in digestive system tumors, and we propose the possible existence of an intratumoral bacteria/mast cell/immune cell axis.
- Specially targeting mast cells, mast cell-derived factors, and the interactions between mast cells and immune cells are expected to improve disease prognosis.

## Open questions

- What is the mechanism by which mast cells secrete relevant mediators in different digestive tracts?
- What is the mechanism of interaction between mast cells and immune cells?
- How do gastrointestinal intratumoral bacteria affect the interaction between immune cells and mast cells?

## Introduction

Mast cells are derived from CD34^+^ and CD117^+^ hematopoietic stem cells in the bone marrow, primarily manifesting as abundant secretory granules in tissues, particularly in areas that interface with the external environment, such as the skin, respiratory tract, and digestive mucosa [1, 2]. Their functions predominantly encompass participation in immune defense, allergic reactions, and tissue repair [3]. Depending on their specific roles, mast cells can be strategically located in various tissue sites, for instance, mast cells found in the gut and skin exhibit distinct phenotypes and functions. In the intestines, their positioning aids in the detection of pathogens and foreign substances; In the skin, it responds quickly to external stimuli and acts as an immune monitor [4, 5]. In addition to the c-kit receptor, which is present from birth, mast cells possess a diverse array of receptors that enable them to detect and respond to multiple ligands [6]. Their significance is underscored by their prominent role in allergic and parasitic reactions, particularly the IgE receptor FcεRI, which is a key receptor in allergic responses [7, 8]. Recently, there has been growing interest in the G protein-coupled receptor X (MRGPRX2) due to its role in recognizing pathogen-associated molecules involved in immune defense, which may led to metamorphic reactions and contribute to chronic inflammatory diseases [9–13]. Additionally, other receptors, such as the IL-33 receptor, which does not rely on mast cell activation, modulate IgE-mediated responses [14]. Upon ligand binding to the mast cell receptor, the mast cell may release mediators such as histamine and proteases (chymases and tryptases) through degranulation, in conjunction with the de novo synthesis of lipid mediators, including arachidonic acid, which can accompany them [15–17]. On the other hand, mast cells can also produce various cytokines and chemokines through degranulation [18]. Furthermore, mast cells secrete exosomes that facilitate the transfer of proteins and nucleic acids, thereby modulating various biological responses [19–21].

Mast cells are characterized by the expression of abundant serine proteases, including tryptase and chymotrypsin, as well as secreted cytokines. Consequently, mast cells can be classified based on the types of protein they expressed: those expressing only tryptase are classified as MCT, while those expressing both tryptase and chymotrypsin are classified as MCTC [22].

According to statistics, in 2018, there were an estimated 4.8 million new cases of digestive system tumors and 3.4 million related deaths worldwide. Of these, gastrointestinal cancers accounted for 26% of global cancer incidence and 35% of all cancer-related deaths [23]. Another systematic analysis revealed that in 2020, the global lifetime risk of developing and dying from gastrointestinal cancer from birth to death was 8.20% and 6.17%, respectively. The risk of developing gastrointestinal cancer is 9.53% for men, while the risk for women is 6.84%; similarly, the risk of death from gastrointestinal cancer is 7.23% for men and 5.09% for women. This means that globally, 1 in 12 people will develop gastrointestinal cancer and 1 in 16 people will die from it [24]. Gastrointestinal cancer is increasingly becoming a significant healthcare burden. Tumors of the digestive system often present with an insidious onset, requiring surgical treatment at the time of diagnosis. Unfortunately, patients frequently experience a poor quality of life post-surgery, and some treatments result in less than outcomes. Therefore, the identification of early-warning biomarkers and new therapeutic for gastrointestinal cancer is urgently needed. Mast cells as sentinel cells in tissues closely associated with chronic inflammation and cancer [25], play a pivotal role in this context. Evidence suggests that mast cells can infiltrate solid tumors, exhibiting a dual role that can either promote or inhibit tumor growth. This paradoxical function makes mast cell a controversial area of research [26–28].

A comprehensive review of the relationship between mast cells in digestive system tumors, as well as their interactions with immune cells and intratumoral bacteria is currently lacking. This paper summarizes the role of mast cells in different digestive tumors. Gaining insight into the molecular mechanisms by which mast cells interact with cancer cells, immune cells, and intratumoral bacteria will aid in identifying strategies to disrupt these interactions and inhibit the growth and proliferation of cancer cells.

## The role of mast cells in different digestive tumors

### Oral cancer

The beginning of the digestive tract is the mouth. The oral cavity is connected to the outside world and later is continued with the pharynx via the pharyngeal isthmus. Organs such as the teeth and tongue are contained in the mouth. The histological structure of the oral mucosa is a complex squamous epithelium which contains a variety of cells. Conseqyently, oral cancer is predominantly squamous cell carcinoma of the oral mucosa and ranks among the most prevalent types of head and neck cancer [29].

Mast cell chymase is a potent enzymatic substance that degrades fibrinogen and fibronectin, thereby promoting tumor progression [30]. A recent study found that mast cells can, secrete chymotrypsin, which activates MIA and MIA2, members of the melanoma-inhibitory active protein gene family in oral squamous carcinoma. This activation promotes angiogenesis and lymphangiogenesis; the underlying mechanism may involve mast cell activation of melanoma inhibitory active motif 2 (MIA2) expression, leading to the upregulation of VEGF-C and -D expression [31]. Additionally, Secernin-1 (SCRN1), a regulatory factor secreted by mast cells, promotes the proliferation, invasion and migration of oral squamous carcinoma cells via TGF-β/Smad3 signaling [32].

Human vascular endothelial cells can modulate mast cell growth by influencing their survival and proliferation, and they can specifically promote the growth and development of MCTC. Research demonstrated that stem cell factor (SCF), vascular cell adhesion molecule VCAM-1 on vascular endothelial cells, and growth factor receptor c-kit along with very late antigen 4 (VLA-4) on mast cells are implicated in the connection within the reaction [33].

Mast cells play a role in the progression of oral squamous carcinoma, however the molecular mechanisms governing their interaction remain unidentified. Alongside chymotrypsin, CCL2 [34] and the extracellular interleukin IL-17F, which exerts a protective effect against squamous cancer of the tongue, are also implicated [35, 36]. Research indicates that patients with squamous oral carcinoma exhibiting a high density of mast cells in the tumor stroma experience prolonged overall survival [37]. This suggests that mast cells may also play a protective role. This phenomenon is paradoxical, and the exact mechanism involved is unclear. However, as far as most recent studies are concerned, mast cells play a major role in promoting the development of oral squamous carcinoma.

Notably, the oral cavity is rich in microorganisms, and further evidence is needed to elucidate the mechanisms of how the oral squamous carcinoma microenvironment, the oral microbial environment, and the mast cells interact with each other.

### Esophagus cancer

Esophageal cancer ranks as the eighth most prevalent malignancy of the digestive system globally and is the sixth leading cause of cancer-related mortality [38]. A projected analysis indicates that in 2020, there will be around 604,000 new instances of esophageal cancer and 545,000 fatalities, with considerable disparities among countries and regions. If current rates persist, esophageal cancer incidences are anticipated to reach 957,000 by 2040, with fatalities escalating to 880,000 in the same year due to population expansion and aging, thereby becoming a global disease burden [39].

Esophageal cancer is classified into two histological types: esophageal squamous cell carcinoma and esophageal adenocarcinoma, with a greater prevalence of esophageal squamous cell carcinoma, and here we focus on the relationship between esophagus squamous cell carcinoma and mast cells. Bioinformatics study revealed that certain individuals with esophageal squamous cell carcinoma exhibited an elevated quantity of mast cells, regulatory T cells, and CD8^+^ T cells. In particular, high density of mast cells in esophagus squamous carcinoma is associated with progression and low postoperative survival in esophagus squamous carcinoma, potentially involving mechanisms such as E2F targets, epithelial-mesenchymal transition, G2/M checkpoints, mitotic spindle dynamics, and the TNFA-NFKB inflammatory pathway [40]. This aligns with another study demonstrating a favorable connection between elevated mast cell density and tumor vascular development, lymph node metastases, tumor infiltration, and progression [41]. It has also been shown in clinical studies that mast cells do not correlate with the prognosis of patients with esophagus cancer [42]. This seems contradictory to the former, on the one hand probably because mast cells release VEGF, histamine, etc. To promote the development of inflammation as well as tumor angiogenesis thus showing promotion of tumor development; On the other hand, the tumor microenvironment also harbors immune cells, such as CD8^+^ T cells and NK cells, which collaborate with mast cells to suppress tumor development. The mechanisms involved remain unclear.

### Gastric cancer

Gastric cancer is a malignant neoplasm arising from the stomach mucosa’s epithelial cells, with risk factors including Helicobacter pylori infection, consumption of nitrite-rich foods, insufficient dietary fiber intake from fruits and vegetables, and advancing age, among others [43]. Global Cancer Statistics 2020 indicate that, despite a reduction in mortality rates in recent decades attributed to early endoscopic screening, there be 1,089,000 new cancer cases and 769,000 deaths worldwide in 2020, positioning it as the fifth most prevalent malignancy and the fourth leading cause of cancer-related fatalities [38]. The progression of gastric cancer is typically influenced by a confluence of factors, including lipid metabolism [44], Immune cell responses [45], Cellular reprogramming within the gastric cancer microenvironment [46], Exosomal non-coding RNAs [47], Tumor-associated fibroblasts [48, 49] and tumor-associated mast cells, etc. [50].

Mast cells also play an important role in gastric cancer. Mast cells secrete tryptase through the activation of protease-activated receptor-2 (PAR2), mitogen-activated protein kinase (MAPK), and the c-kit receptor；The density of tryptase-positive mast cells has a substantial correlation with the density of lymph node metastases in primary gastric cancer tissue and the prognosis of gastric cancer patients [51, 52]. Gastric cancer tissues exhibiting elevated levels of IL-17AmRNA and IL-17A contained a greater abundance of anti-tumor mast cells and NK cells, and lower tumor-promoting macrophages [53], the mechanisms involved require further study. Interestingly, Gastric cancer cells can stimulate mast cells to secrete IL-17A through degranulation of the PI3K-AKT pathway by secreting adrenomedullin (ADM), hence facilitating tumor growth [54].

The interaction between mast cells and IL-33 in the gastrointestinal system is particularly significant [55]. IL-33 initiates a type 2 immune response by activating mast cells or other immune cells, including T cells; Mast cells can release IL-2 via the IL-33 and ST2 pathway, which facilitates the differentiation of CD4^+^ T cells into ICOS^+^ regulatory T cells while suppressing the activity of CD8^+^ T cells, thus fostering tumor progression [56], but the mechanism of interaction of these molecules between mast cells and Treg and whether there is a lack of studies on other immune cells is ambiguous.

Mast cells can augment pertinent pathways to facilitate tumor progression. Mast cells, as sentinel cells within the tumor microenvironment, can engage in diverse immune responses via many surface receptors, such as the immunosuppressive molecule programmed death ligand PD-L1 [57]. Research indicates that mast cells in gastric cancer can suppress T cell proliferation through the TNF-α-PD-L1 axis, thereby facilitating tumor growth [58, 59].

The interaction between mast cells and T cells in gastric cancer is notably intimate, and the mechanisms that dictate tumor development or dormancy through the release of specific signals are intricate and warrant further experimental investigation [28].

### Small intestine cancer

Small intestinal cancer is an exceedingly rare malignant neoplasm that arises in the duodenum, jejunum, and ileum. Owing to its unique location, the majority of cases are diagnosed at an advanced stage, resulting in a prognosis that is frequently worse than that of other malignant neoplasms, such as colorectal cancer [60–62]. Consequently, given this trait, it is essential to enhance the comprehension of small intestinal cancer.

The intestinal tract is rich in mast cells, which on the one hand have immunological, reparative and homeostatic functions; On the other hand, when activated inappropriately by allergic mediators, they can show disturbed pathogenic mechanisms, leading to various intestinal manifestations [63, 64]. Investigating the role of mast cells in the small intestine may provide therapeutic targets to reduce mortality from small bowel cancer.

The intestinal tract, as a lumen for digestion, absorption and waste elimination, is rich in mucosal and connective tissues. Therefore, mast cells can be divided into mucosal mast cells (MMCs) and connective tissue mast cells (CTMCS) [65, 66]. Meanwhile, based on molecular expression analysis, mast cells expressing mouse mast cell protease 1 (mMMp1), mMMP2, mMMP6, and mMMP7 are classified as lymphocyte-dependent MMCs; those expressing tetrameric class trypsin mMCP6, chymase mMCP4, and elastase mMCP5 are categorized as non-lymphoid-dependent CTMCs, and their expression in expression in various tissues had significant differences [67, 68].

Gastrointestinal polyps, which represent a heterogeneous group of lesions that can be classified as either cancerous or non-cancerous, play a significant role in the progression of cancer [69]. In the context of small bowel polyps, mast cells can facilitate the progression to small bowel cancer in a tumor stage-specific and cytokine-dependent manner [70]. In the mouse model of polyposis with hereditary deletion of the APCA gene, the levels of IL-10, IL-13, and IL-33 were elevated, and the population of type 2 innate lymphoid cells (ILC2s) was enriched, Meanwhile, mast cells exhibited a localized expansion in this context. Mast cells attach to transformable epithelial cells and express mMCP2, along with the connective tissue mast cell (CTMC) protease mMCP6, albeit to a lesser extent. The survival of these mast cells is strictly dependent on IL-10 derived from T cells; In the absence of IL-10-expressing T cells, the development of small intestinal polyps is significantly delayed [71]. Interestingly, despite a massive expansion of mast cell in polyposis-prone mice, overexpression of IL-10 by T cells does not lead to an increase in polyp formation. This suggests a dual role for IL-10: while it promotes mast cell expansion, it also enhances T cell immunosurveillance and supports the recovery of regulatory T cells, thereby demonstrating a protective function [72, 73]. The intricate biological relationship between IL-10 and polyposis necessitates further experimentation to elucidate the mechanisms underlying their interaction. Notably, as polyps become aggressive, there is an expansion of mMCP5^+^/mMCP6^+^ CTMCs in the interstitium and at the invasive tumor borders. The ablation of mMCP6 expression can alleviated, polyposis, indicating a potentially significant role for CTMC in the transformation of small bowel polyps into small bowel cancer.

IL-33 functions as a pro-inflammatory signal by binding to ST2 surface receptors located on mast cells, type 2 intrinsic lymphoid cells (ILC2s), and Th2 cells, leading to the production of type 2 cytokines such as IL-4, IL-5, and IL-13, which elicits a corresponding immune response [74–76]. A recent study has demonstrated that in a mouse model of BCR-ABL oncogene-dependent chronic granulocytic leukemia CML, the disease induces an inflammatory response in the gut. This response results in the production of the inflammatory mediator IL-33 by stressed epithelial cells, which then binds to ST2 receptors on type 2 intrinsic lymphoid cells, prompting them to produce IL-9. IL-9, in turn, promotes the expansion and remodeling of small intestinal mucosal mast cells [77]. This finding highlights an intriguing relationship between ILC2s and mast cells in mouse polyposis, although the exact mechanism requires further investigation.

Moreover, the small intestine, being the primary site for digestion and absorption, often induces significant psychological distress in patients when it becomes cancerous. Excessive psychological stress can enhance mast cell activation via the adrenocorticotropin-releasing hormone-mast cell axis, resulting in clinical symptoms such as stomach pain [78]. At the same time, it affects the function of the Bruch’s glands in the duodenum, which in turn affects the intestinal flora, particularly resulting in a decrease of Lactobacillus spp. bacteria, which finally causes intestinal inflammation and diminished immunity [79]. The cycle may induce positive feedback in the progression of small bowel cancer.

### Pancreatic cancer

Pancreatic cancer, a type of cancer with a very poor prognosis, is known as the “king of cancers”. According to statistics, the total number of pancreatic cancer cases is expected to rise from 458,918 in 2018 to 814,235, an increase of about 61.7% [80, 81]. Among the various types of pancreatic cancer, pancreatic ductal adenocarcinoma is the type of pancreatic cancer with the highest incidence, the highest aggressive and the highest mortality rate; when KRAS is mutated, the oncogenes occur in the order of CDKN2A, TP53 and SMAD4, culminating pancreatic ductal adenocarcinoma [82, 83].

Mast cells significantly influence the development of solid tumors and inflammation. They contribute to tumor progression and the transition from pancreatitis to pancreatic cancer. Mast cells promote the invasive behavior of pancreatic ductal adenocarcinoma by increasing levels of IL-13, tryptase, VEGF, platelet-derived growth factor, and fibroblast growth factor-2, all while maintaining cell membrane integrity [84, 85]. During pancreatitis, mast cells accumulate in the inflammatory fibrotic areas of the pancreas and in the surviving acinar tissue, accelerating inflammatory invasion and pancreatic tissue remodeling by degranulation and release of inflammatory mediators [86]. The differences in mast cell membrane integrity observed between pancreatitis and pancreatic cancer suggest that these cells may aid tumor progression by producing pro-tumor factors [87].

### Liver cancer

Liver cancer, a malignant tumor prevalent worldwide, ranks among the top five causes of cancer-related deaths, with its incidence increasing annually [88, 89]. Primary liver cancers are classified according to their tissue type and primarily include hepatocellular carcinoma (HCC), intrahepatic cholangiocarcinoma (ICC), and Combined hepatocellular-cholangiocarcinoma(ChCC-CCA). Among these, hepatocellular carcinoma constitutes the largest proportion, accounting for approximately 75–85%, while intrahepatic cholangiocarcinoma represents about 10–15% [90–92].

Resting mast cells gather in the liver primarily in small numbers in the conjoined area of the hepatic lobules and near the hepatic blood sinusoids, suggesting that they are not exclusively linked to inflammation and damage in the liver [93]. Only under specific conditions, mast cells receive specific signals, and thus transitioning from a resting state to an activated state and migrating from the bone marrow [94]. Furthermore, after originating from CD34^+^ multipotent stem cells in hematopoietic organs, mast cells consistently express c-kit during their development, and their role in the liver is controversial [95, 96].

In the human hepatocellular carcinoma cell line HuH-6, mast cells stimulate the activation of caspase-3 and poly (ADP-ribose) polymerase (PARP) by releasing histamine and degranulating, this resulted in the down-regulation of COX-2, survivin, and β-catenin expression in HuH-6 cells, leading to decreased activity and proliferation. In contrast, in the HA22T/VGH cell line, there was an upregulation of survivin and β-catenin [97].

Another study has demonstrated that the infiltration of various immune cells among different metabolic subtypes of hepatocellular carcinoma is closely associated with clinical prognosis. For instances, patients with hepatocellular carcinoma characterized by a high infiltration of resting mast cells exhibit a shorter survival period in the glycolytic subtype [98, 99].

Currently, the prognosis and recurrence rates for hepatocellular carcinoma patients can be estimated by evaluating the density, distribution, and structure of mast cells [100, 101]. In the future, only further experimental studies on mast cells and metabolic subtypes of tumors will provide more precise prognostic predictions and molecular targets for hepatocellular carcinoma treatment.

### Cholangiocarcinoma

Cholangiocarcinoma represents a malignant neoplasm that can occur in any section of the bile ducts, classifying into intrahepatic and extrahepatic varieties. Primarily, it presents as adenocarcinoma, with other histological types appearing infrequently [102]. In regions where the disease is endemic, liver fluke infection significantly contributes to bile duct pathology. Conversely, in non-endemic regions, chronic inflammatory changes stemming from biliary stones and primary sclerosing cholangitis predominantly facilitate cholangiocarcinoma development [103].

Both cholangiocarcinoma tumor cells and bile duct endothelial cells have the capacity to secrete SCF. SCF functions as a ligand for the c-KIT receptor on mast cells. This interaction recruits mast cells to the tumor microenvironment and triggers their release of histamine. This cascade accelerates both cholangiocarcinoma progression and angiogenesis [104]. Thus, exploring mast cell involvement in cholangiocarcinoma may unveil new therapeutic targets for its treatment.

In cholangiocarcinoma, tumor-associated fibroblast-derived SCF recruits mast cells. The transhepatic production of biliary N, N-dimethyl-1,4-phenylenediamine (DMPD) mediates through the activation of G protein-coupled receptor subtype 2 (MRGPRX2)-GαQ signaling in mast cells. This process induces the release of histamine (HA), platelet-derived growth factor B subunit (PDGF-B), and angiopoietin 1/2 (ANGPT1/2) from mast cells [105]. PDGF-B and ANGPT1/2 drive both angiogenesis and lymphangiogenesis. Histamine stimulates cholangiocarcinoma proliferation via the HRH1-GαQ signaling pathway. Simultaneously, it activates the HRH2-GαS signaling pathway, enhancing the secretion of SCF from tumor-associated fibroblasts, thus establishing positive feedback between SCF and histamine. Furthermore, cholangiocarcinoma releases the exosomal miR-182/183-5p into bile, targeting hydroxyprostaglandin dehydrogenase in both cholangiocarcinoma and mast cells, leading to an increased release of prostaglandin E2 and VEGF-A. Prostaglandin E2 promotes cholangiocarcinoma stemness through PTGER1 activation, while VEGF-A encourages angiogenesis [106]. Notably, research indicates that Tryptase-positive tumor-infiltrating mast cells correlate positively with anti-tumor CD8^+^ T cells, contributing to improved overall patient survival [107].

In conjunction with existing studies, mast cells in cholangiocarcinoma can exhibit a dual role. On the one hand, probably because of the different mast cell subtypes, for example, mast cell subtype 1 has anti-tumor activity while mast cell subtype 2 has pro-tumor activity [108, 109]. On the other hand, it may be related to different metabolic subtypes in cholangiocarcinoma.

### Colorectal cancer

The incidence and mortality rates of colorectal cancer (CRC) are gradually increasing worldwide. Notably, early exposure in birth cohorts has influenced the epidemiology of colorectal cancer, leading to a rise in early-onset colorectal cancer (EOCRC, age <50 years). This phenomenon may be related to stem cell activity in the colorectum of early-onset patients as well as retrograde mechanisms [110–112]. Consequently, it has become particularly important to study the immunity, exposure factors, and associated causative genes in the development of colorectal cancer. Recent research has shown that mast cells are associated with a variety of cancers, and their role in the progression of colorectal cancer has been increasingly recognized [108]. Therefore, this section focuses on the role of mast cells in colorectal cancer.

Single-cell analyses, spatial and transcriptomics have revealed that mast cells play a more critical role in colorectal cancer. Mono cytological analysis has identified two distinct subpopulations of mast cells within tumors, differentiated by their expression of TNF and VEGF-A. Mast cells exhibiting high levels of TNF and VEGF-A are characterized as antitumorigenic, whereas those with lower expression levels are deemed pro-tumorigenic [113]. Another single-cell analysis showed that although the density of mast cells in colorectal cancer was lower than in normal tissue, resting mast cells with high expression of chymase1 (CAM1) were activated to mast cells with high expression of TPSAB1, CPA3, and KIT [114]. These findings collectively highlight the complex and dynamic role of mast cells in the tumor microenvironment of colorectal cancer.

In a study supporting the role of mast cells in promoting colorectal cancer development, several mechanisms have been identified. Firstly, mast cells can release angiogenic factors such as VEGF-A, CXCL-8, MMP-9, FGF-2, PDGF, and IL-6, as well as lymphangiogenic factors like VEGF-C and VEGF-D, and matrix metalloproteinases (MMP), all of which contribute to the promotion of colorectal cancer [115, 116]. Secondly, chronic inflammation, a known driver of cancer development and progression, involves mast cells as key inflammatory cells [117, 118]. Mast cells utilize 5-lipoxygenase, an enzyme that acts on arachidonic acid, to limit intestinal epithelial cell proliferation and mobilize myeloid-derived suppressor cells (MDSCs). In the colorectal polyposis APC (Δ468) mouse model, specific deletion of the 5-lipoxygenase gene significantly attenuates polyp development and MDSC function [119]. Furthermore, in a mouse model deficient in the NFE2L3 transcription factor, mast cell activity can be regulated through the upregulation of RAB27A, which enhances IL-33 activity and mediates the transition between inflammation and cancer [120, 121].

In colorectal cancer patients classified as stage II and III who have not received adjuvant chemotherapy, high-density mast cells are associated with improved survival rates. Conversely, following adjuvant chemotherapy, an increased density of mast cells correlates negatively with recurrence-free survival, overall survival, and CD8^+^ T-cell infiltration. This inverse relationship may be attributed to the varying stages of leukocyte infiltration and the activation and transformation of mast cells in colorectal cancer [122, 123]. Furthermore, mast cells secrete cystatin C, which induces endoplasmic reticulum stress and activates the unfolded protein response (UPR) specifically in colorectal cancer cells, thereby inhibiting cancer progression without affecting normal colorectal tissues [124].

It is worth highlight that the effect of mast cells in colorectal cancer is controversial, with different immune types altering mast cell activity and status and vice versa [125]. Most of the current stage of mast cell and colorectal cancer research focuses on the role of related transmitters in the relationship between mast cells and colorectal cancer, but the specifics are not yet clear.

**Fig. 1:**
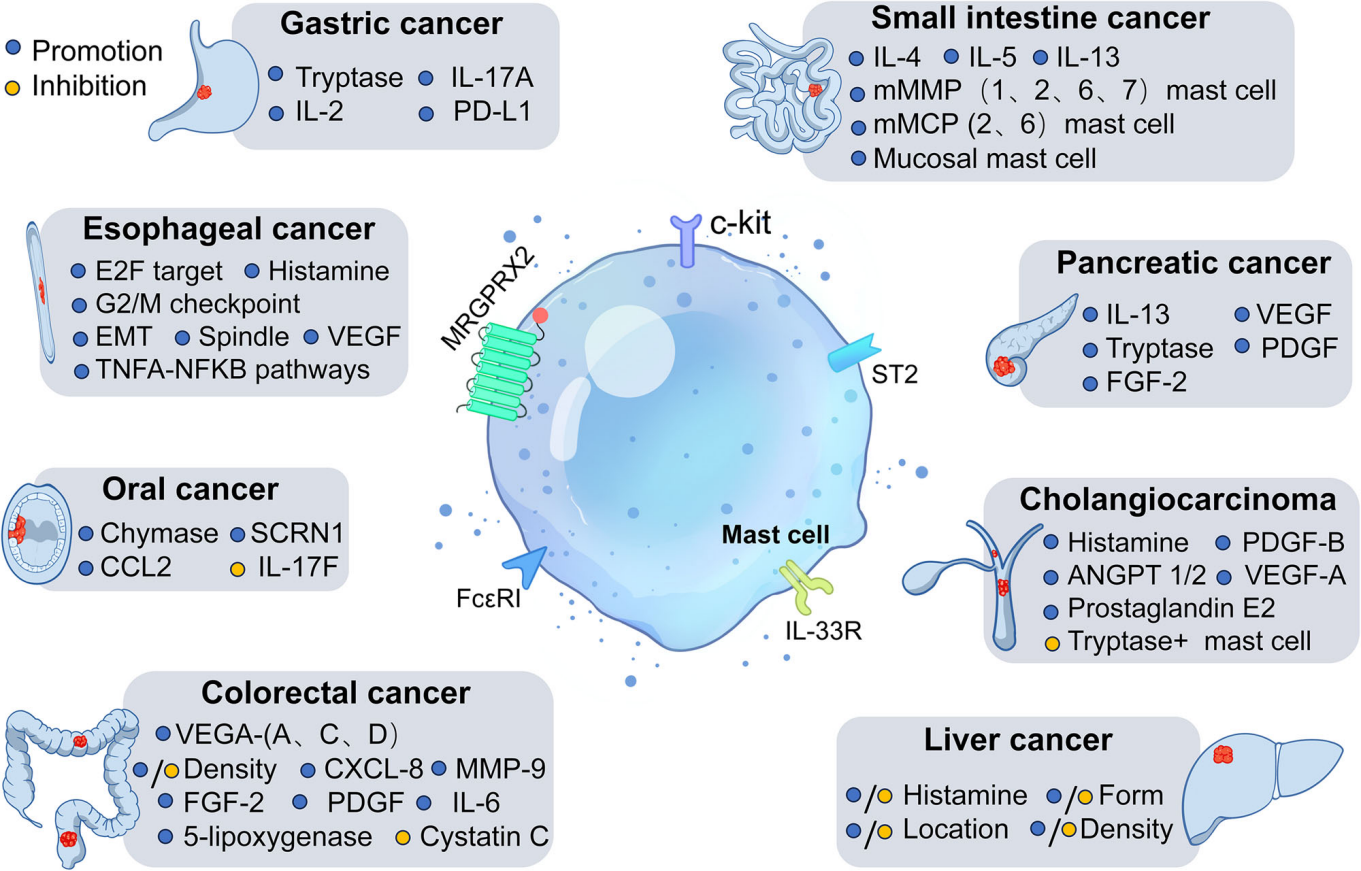
The role of mast cells in digestive system tumors. This figure illustrates the diverse roles of mast cells in digestive system tumors, including their ability to either promote or inhibit tumor growth. Mast cells exert their effects by releasing substances such as histamine, tryptase, and chemotactic factors. The figure uses color-coded circles to represent different effects: blue indicates tumor-promoting functions, while yellow represents tumor-inhibiting functions.

In summary, mast cells display a spectrum of functions in various digestive system tumors (Fig. 1). Present observations suggest that mast cells primarily serve as promoters within these tumors. Elucidating the intricate mechanisms underpinning their roles necessitates more profound investigation.

## The interaction of mast cells and tumor infiltration immune cells

**Fig. 2:**
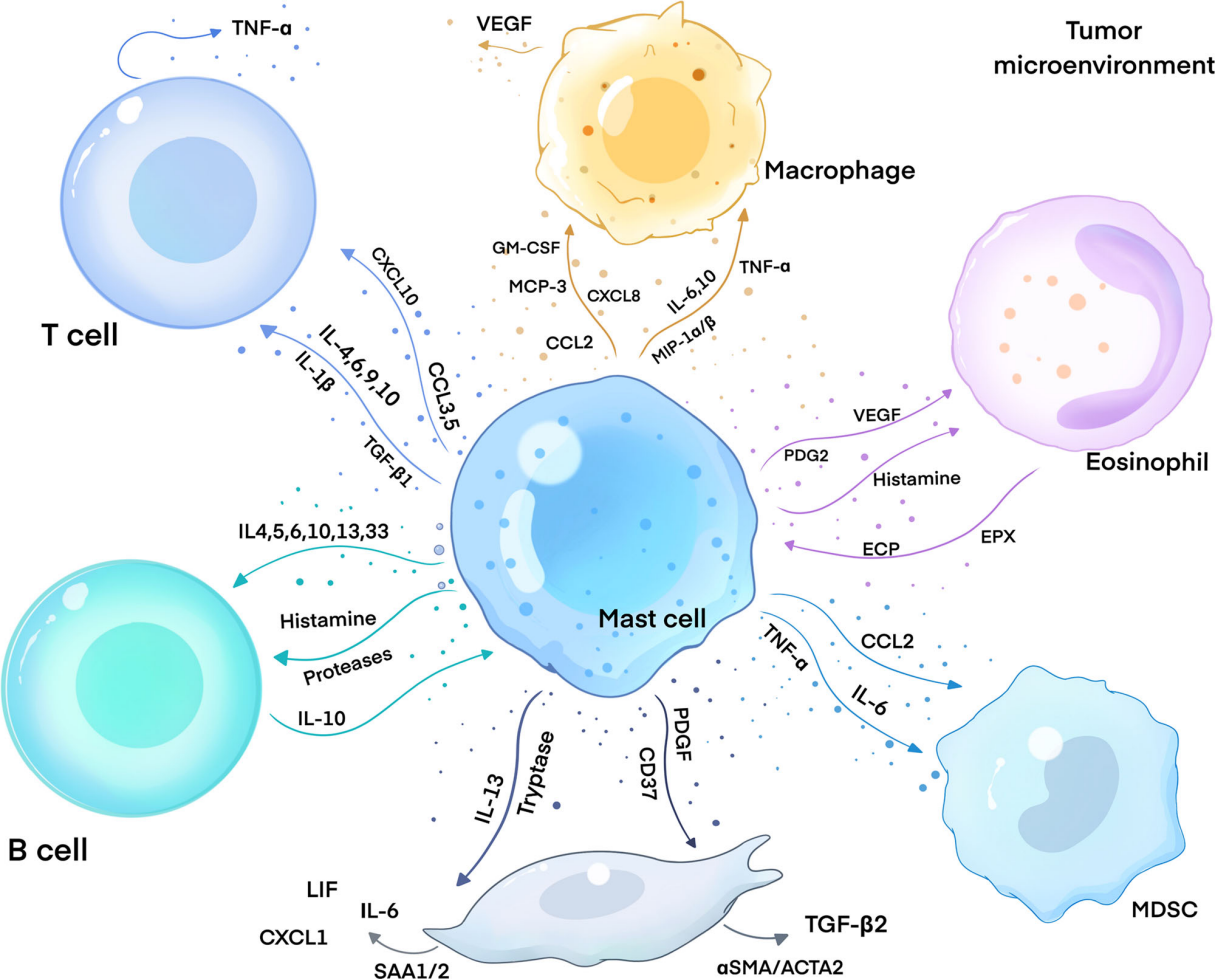
The interaction of mast cells and tumor infiltration immune cells. Mast cells exhibit complex interactions with various infiltrating immune cells within the tumor microenvironment of gastrointestinal cancers. By releasing an array of bioactive substances, including histamine, tryptase, chemokines, and cytokines, mast cells modulate the recruitment of immune cells and the secretion of related mediators, thereby influencing tumor progression and the remodeling of the tumor microenvironment.

The presence of infiltrating immune cells in the tumor microenvironment complicates the occurrence and development of tumors, particularly within the digestive system. Notably, there are specific interactions among immune cells in this environment. In this section, we primarily focus on the interactions between mast cells and various immune cells, including fibroblasts, macrophages, T cells, B cells, myeloid-derived suppressor cells, and eosinophils within the digestive system’s tumor microenvironment (Fig. 2). The primary aim is to provide a reference for future basic and clinical research on the microenvironment of digestive tract tumors.

### Mast cells and fibroblasts

The interplay between mast cells and tumor-associated fibroblasts within the tumor microenvironment has been shown to significantly contribute to tumor progression [126, 127]. Previous studies indicate that in pancreatic cancer, activated mast cells can stimulate the proliferation of fibroblasts, specifically pancreatic stellate cells, through the secretion of IL-13 and tryptase. This interaction leads to the release of TGF-β2 from fibroblasts, promoting fibrosis in pancreatic cancer tissue and consequently diminishing the efficacy of antitumor therapies [128]. Notably, a recent study demonstrated that extracellular vesicles (EVs) derived from BAG6-deficient pancreatic cancer cells activate mast cells via the IL33/IL1RL1 pathway. These activated mast cells, in turn, induce tumor proliferation and modify the tumor microenvironment by secreting factors such as PDGF, CD73, and IL-13. A critical outcome of this interaction is the polarization of fibroblasts into inflammatory cancer-associated fibroblasts (iCAFs), which exhibit upregulation of genes including IL-6, SAA1/2, CXCL1, LIF, and αSMA/ACTA2, ultimately contributing to tumor progression [129].

### Mast cells and macrophages

In gastrointestinal tumors, mast cells and tumor-associated macrophages interact through a complex network of cytokine and chemokines to regulate tumor progression. Pro-inflammatory M1 macrophages and immunosuppressive M2 macrophages play distinct roles, with M2 macrophages primarily advancing tumors by promoting angiogenesis and immunosuppression. In gastric cancer, chemokines such as CCL2 and CXCL8 secreted by mast cells recruit macrophages and facilitate their polarization toward the M2 phenotype. Polarized M2 macrophages subsequently release VEGF and IL-6, which supports tumor progression [130]. Additionally, cytokines such as IL-6, IL-10, and TNF-α secreted by mast cells can induce M2 polarization of macrophages, thereby allowing carcinoma to evade immune surveillance [131]. Importantly, IL-33 secreted by gastric cancer tissue can activate ST2^+^ mast cells, which then release granulocyte-macrophage colony-stimulating factor (GM-CSF), monocyte chemoattractant protein-3 (MCP-3), and macrophage inflammatory proteins 1α and 1β (MIP-1α and MIP-1β). These factors promote macrophage activation, thereby enhancing tumor cell proliferation and resistance to apoptosis [132].

### Mast cells and T cells

In the tumor microenvironment, mast cells release histamine and various cytokines, including IL-4, IL-6, IL-9, IL-10, IL-1β, and TGF-β1. Notably, IL-1β and histamine promote T cell differentiation toward the Th2 phenotype, whereas IL-2 and TGF-β1 facilitate the development of regulatory T cells. This regulatory mechanism has been extensively studied in gastric cancer and demonstrates similar roles across other gastrointestinal cancers [56, 133, 134]. Furthermore, mast cells secrete chemokines such as CXCL10, CCL3, and CCL5, which recruit CD8^+^ and CD4^+^ T cells to the tumor microenvironment. The secretion of TNF-α by these T cells can further modulate T cell activity, thereby enhancing anti-tumor responses [135]. Importantly, interactions between mast cells and T cells can, in certain contexts, inhibit the anti-tumor functions of T cells, potentially through the PD-L1 pathway or via TGF-β factors, thus facilitating tumor immune evasion. This mechanism has been observed in colorectal cancer and may be associated with the complexity of the microbiota within the colorectal environment [125].

### Mast cells and B cells

Mast cells can pass through a variety of cytokines (IL-4, 5, 6, 10, 13, 33, B-lymphocyte stimulants), Membrane-bound receptors and ligands (CD40L/CD40, OX40L/OX40, CD70L/CD70, CD30L/CD30) and granule products (histamine and proteases) interacting with B lymphocytes. On the other hand, B cells can crosstalk with it via IL-10. These interactions promote the proliferation and survival of B lymphocyte [136, 137].

### Mast cells and myeloid-derived suppressor cell

In a mouse model of liver cancer, activated mast cells can recruit myeloid-derived suppressor cells (MDSCs) through the CCL2/CCR2 axis and induce them to secrete IL-17, which further recruits Tregs [138]. In the transgenic APCΔ468 mouse model of colon cancer, mast cells facilitate the migration of MDSCs by producing 5-lipoxygenase. Beyond these cellular mediators, the direct interaction between mast cells and MDSCs via the CD40L/CD40 axis can enhance the production of TNF-α, IL-6, and CCL2, thereby further activating MDSCs [119, 139].

### Mast cells and eosinophils

Eosinophils have been identified in the tumor microenvironment of four types of digestive tumors: oral cancer, esophageal cancer, gastric cancer, and colon cancer [140–143]. Given that eosinophils and mast cells share similar developmental and functional patterns, studying their relationship within the tumor microenvironment is of considerable significance. Currently, it is widely accepted that mast cells can secrete VEGF, which recruits eosinophils into the tumor microenvironment. Additionally, mast cell its mediate eosinophil migration through the histamine/H4R and PDG2/CRTH2 [144, 145].

Eosinophils also release substances that influence mast cells. The release of eosinophilic cationic protein (ECP) and eosinophilic peroxidase from eosinophils can enhance the immune response in mast cells, potentially leading to chronic inflammation. Furthermore, eosinophils also can produce the substance P amide and myelin basic protein, and they can interact with the MAS-associated G-protein-coupled receptor-x2 (MRGPRX2) on mast cells [146, 147].

## Intratumoral bacteria in digestive system tumors may interact with mast cells and immune cells

In previous views, sterile organs and tissues, such as the pancreas and liver, were considered to be devoid of microbial populations. Nowadays, there is increasing evidence that sterile organs also contain different microbial populations [148]. How the microbiome affects tumors has long been a hot topic of research. Recent literature suggests that the microbiome within a tumor is inextricably linked to the tumor [149]. Furthermore, the characterization of the intratumoral microbiome with respect to certain immune cell populations in the tumor microenvironment has been well described. Mast cells, which are among the immune cell associated with tumors, engage in crosstalk with other immune cells [150]. Consequently, we speculate that in digestive system tumors there is a relevant crosstalk between intratumoral bacteria and immune cells and mast cells. Despite the correlation, a causal relationship has yet to be established. This section aims to provide a foundational reference for the relationship between intraluminal bacteria of the digestive system and immune cells, including mast cells, in future studies of gastrointestinal tumors.

### Pancreatic cancer

Porphyromonas gingivalis has been identified in cases of pancreatic cancer and is known to specifically upregulate genes associated with inflammatory responses and neutrophil chemotaxis, including Cxcl2, Cxcr2, IL17f, S100a8, and S100a9, as reported in a study. This upregulation results in the secretion of neutrophil chemokines that promote the infiltration of tumor-associated neutrophils, thereby facilitating the progression of pancreatic cancer. Additionally, Porphyromonas gingivalis enhances the secretion of neutrophil elastase by tumor-associated neutrophils, further supporting the advancement of pancreatic cancer [151].

Neutrophil elastase can participate in human inflammatory response, remodel the tumor microenvironment, promote invasive metastasis and malignant development of tumor cells, and promote cancer drug resistance. In addition, neutrophil elastase can stimulate and activate mast cells, macrophages and other cells [152].

In addition to the bacteria mentioned above, fungi within pancreatic cancer (e.g., Streptococcus spp. and Malassezia spp.) are also play a role in its the progression of pancreatic cancer. Studies have shown that fungus-derived components in pancreatic cancer can induce cancer cells to secrete IL-33 via the dectin-1-mediated Src-Syk-CARD9 pathway. This process recruits and activating Th2 and ICL2 cells, which, in turn, can facilitate the progression of pancreatic cancer through the secretion of cytokines such as IL-4 and IL-13 [153]. Notably, IL-33R was also present on both mast cells and eosinophils, and there exists some crosstalk between these two cell types, but this was not taken into account in this study.

We propose that a Porphyromonas gingivalis/neutrophil/mast cell axis may be exist in pancreatic cancer, along with a Fungus/IL-33/mast cell axis, a Fungus/mast cell/Th2 axis, and the crosstalk axis involving IL-33, eosinophils, and mast cells. This hypothesis requires further experimental validation.

### Colorectal cancer

Fusobacterium nucleatum has been demonstrated to play a significant role in colorectal cancer [154]. This bacterium secretes various cytokines, including IL-6, IL-8, TNF-α, and MCP-1, which induce chemotaxis and activate tumor-associated neutrophils. The activation of these neutrophils results in an increase in reactive oxygen species, thereby promoting the carcinogenesis of colorectal tissues. This is embodied in two aspects: On the one hand, Fusobacterium nucleatum can contribute to colorectal cancer through a TLR4-dependent mechanism. Specifically, it promotes the polarization of monocytes to M2 macrophages within the colorectal tumor microenvironment via the IL-6/STAT-3/c-MYC pathway, which facilitates angiogenesis and further supports carcinogenesis [155]. On the other hand, lipopolysaccharide from it induces macrophages to secret IL-6, IL-8, IL-1β, TNF-α, PGE2, and MMP-9. As illustrated in the “Mast cells and macrophages” section, IL-6 secreted by macrophages can influence mast cells, thereby maybe producing additional effects that contribute to the tumor microenvironment.

Bacterial colonies not elucidated in detail in colorectal cancer can induce IL-17 production, which recruits B cells to infiltrate into the tumor tissue, ultimately leading to colorectal cancer [156]. As shown in mast cells and B cells, B cells can affect mast cells via IL-10.

We propose the existence of a fusobacterium nucleatum/tumor-associated neutrophil/mast cell axis, a fusobacterium nucleatum/tumor-associated macrophage/mast cell axis, and an intratumoral bacterial/B-cell/mast cell axis in the development of colorectal cancer.

### Gastric cancer

The development of gastric cancer is closely associated with exogenous infection by Helicobacter pylori (Hp), primarily through the induction of chronic inflammation. This chronic inflammation accelerates DNA replication and impairs the mechanisms responsible for DNA damage repair, resulting in an imbalance and inactivation between the ratio of oncogenes and tumor suppressor genes. Consequently, these molecular alterations contribute to the progression and development of gastric cancer [157].

The major pro-inflammatory factor in Hp is neutrophil activating protein (NAP), which is inextricably linked to the development of gastric cancer. NAP activates neutrophils to produce reactive oxygen species (ROS) and recruit neutrophils to adhere to the gastric epithelium. In addition, NAP induces the release of β-glucosaminidase, IL-6 and histamine from mast cells, which promotes the inflammatory cascade [158]. From this, we propose that in gastric cancer, there may be HP/mast cell axis, HP/mast cell/T/B axis, and HP/mast cell/macrophage axis.

**Fig. 3:**
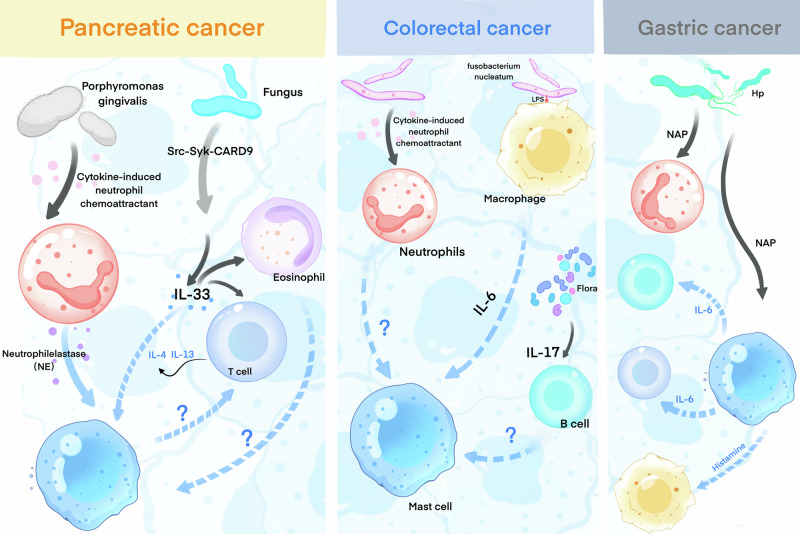
Intratumoral bacteria and possible immune regulation of mast cell in digestive system tumors. This figure illustrates the complex interaction mechanisms between intratumoral bacteria, immune cells, and mast cells in pancreatic cancer, colorectal cancer, and gastric cancer. Intratumoral bacteria regulate immune cell activity and mast cell function through direct interactions or the secretion of metabolic products, thereby influencing the tumor immune microenvironment and tumor progression. In the figure, question marks represent potential connections that remain unexplored but are hypothesized to exist.

We reveal a correlation between intratumoral bacteria and mast cells in pancreatic, colorectal, and gastric cancers (Fig. 3). However, the precise mechanisms remain to be clarified. Additionally, research on intratumoral bacteria in esophageal, oral cavity, liver, and biliary tract cancers highlighted disparities in microbiome composition and immune cell infiltration across various gastrointestinal tumors [159–163]. Nonetheless, the specific molecular interactions between bacterial strains and immune cells require further exploration.

## Conclusions

In summary, mast cells play diverse roles in various tissues of the digestive system tumors (Fig. 1). Current research predominantly focuses on the relationship between mast cells and tumor-related mediators. However, several aspects remain inadequately explored, including the relevant pathways, mast cell subsets, activation criteria, tissue localization, and the crosstalk between mast cells and other immune cells. Based on existing studies, the therapeutic targeting of mast cells in tumors can be categorized into four main strategies: targeting mast cell-derived mediators [164], inhibiting mast cell activation [165, 166], silencing mast cells [167, 168], and reducing the number of mast cells [96]. Mast cells, often considered controversial, exhibit particularly complex mechanisms within the high metabolism and microenvironment of tumors. To gain a deeper understanding of the role of mast cells in digestive system tumors, it is essential to conduct research on specific populations of mast cells in particular digestive system cancers. Additionally, we propose investigating the crosstalk between mast cells and other immune cells, as well as the potential immune regulatory interactions between intra-tumoral bacteria in the gastrointestinal tract and mast cells. Such research could provide new insights into the clinical treatment of gastrointestinal tumors.

## Data Availability

The materials in this study are available to provide by corresponding authors on reasonable request.
